# Early peri-implant crevicular fluid interleukin-1β as a potential predictor of peri-implant diseases: a 12-month prospective study

**DOI:** 10.3389/fmed.2026.1786515

**Published:** 2026-03-09

**Authors:** Su-Wei Fu, Juan Zhao, Fei Yang, Zhi-Yan Shi, Shen Li, Dong-Liang Xu

**Affiliations:** Department of Stomatology, Henan Provincial People’s Hospital, People's Hospital of Zhengzhou University, Zhengzhou, Henan, China

**Keywords:** interleukin-1β, interleukin-6, peri-implant diseases, peri-implant mucositis, peri-implantitis, tumor necrosis factor-α

## Abstract

**Objective:**

Peri-implant disease is a common complication of implant-supported rehabilitation. This study aimed to explore the association between inflammatory cytokine levels in peri-implant crevicular fluid (PICF) and the development of peri-implant diseases.

**Methods:**

Patients who underwent dental implant surgery at the Department of Stomatology, Henan Provincial People’s Hospital between July and December 2024 were prospectively enrolled and received routine postoperative oral care. At 3 months postoperatively, levels of interleukin-1β (IL-1β), interleukin-6 (IL-6), and tumor necrosis factor-α (TNF-α) in PICF were measured. The incidences of peri-implant diseases at 12 months postoperatively were determined. Patients were grouped according to the presence or absence of peri-implant diseases, and the associations between PICF inflammatory cytokines and peri-implant diseases were analyzed.

**Results:**

A total of 111 patients (157 implants) were screened; 65.8% (73/111) were male, with a mean age of 60.6 ± 7.1 years. At 3 months postoperatively, median PICF levels of IL-1β, IL-6, and TNF-α were 20.8 (15, 23.4), 3.7 (2.6, 5.0), and 13.7 (11.8, 16.0) pg./mL, respectively. At 12 months postoperatively, the incidences of peri-implant mucositis and peri-implantitis were 14.4% (16/111) and 6.3% (7/111), respectively. In univariate logistic regression analyses, both IL-1β and IL-6 were suggested as potential predictors of peri-implant mucositis and peri-implantitis (all *p* < 0.05). In multivariate logistic regression analyses, only IL-1β remained a potential predictor (both *p* < 0.05). For IL-1β > 25 pg./mL, the receiver operating characteristic curve analysis within this dataset yielded areas under the curve of 0.833 (*p* < 0.0001) and 0.804 (*p* = 0.0094) for peri-implant mucositis and peri-implantitis, respectively, suggesting possible discriminatory ability.

**Conclusion:**

Elevated IL-1β levels in PICF at 3 months postoperatively may serve as a potential predictor of peri-implant disease at 12 months after implant placement. These findings will need to be validated in larger, multicenter studies in the future.

## Introduction

Peri-implant diseases, including peri-implant mucositis and peri-implantitis, are among the most common biological complications affecting the long-term success of dental implants (1, 2). Although substantial progress has been made in recent years in understanding their pathogenesis and developing therapeutic strategies, early clinical identification of peri-implant diseases remains challenging (3–9). Currently, diagnosis mainly relies on clinical and radiographic parameters such as probing depth, bleeding indices, and radiographic bone loss (10, 11). Recently, Önder and Alpaslan (12) reported in a prospective cohort study that peri-implant phenotype, particularly a thin biotype, was associated with greater peri-implant disease severity. However, these indicators largely reflect established tissue inflammation or bone destruction and are insufficient for the timely detection of the early inflammatory changes that precede disease onset.

Studies have shown that peri-implant disease is essentially a plaque biofilm–induced condition mediated by the host immune–inflammatory response, in which multiple proinflammatory cytokines play critical roles in the initiation of inflammation and subsequent tissue destruction (8, 10, 11, 13, 14). The roles of inflammatory mediators such as interleukin-1β (IL-1β), interleukin-6 (IL-6), and tumor necrosis factor-*α* (TNF-α) in periodontitis have been well established and are closely associated with disease severity and progression (6, 7, 15–19). Meanwhile, significantly higher levels of IL-6, IL-1β, TNF-α, and active metalloproteinase-8 were also detected at peri-implantitis and periodontitis sites than at healthy sites (15). However, compared with periodontitis, research on peri-implant diseases has predominantly focused on disease diagnosis or correlation analyses, and prospective studies evaluating the predictive value of peri-implant crevicular fluid (PICF) inflammatory cytokines for the development of peri-implant disease remain relatively limited.

Moreover, although periodontal tissues and peri-implant tissues share certain similarities in anatomical structure and immune responses, substantial differences exist in soft and hard tissue architecture, biological sealing mechanisms, and the local immune microenvironment. Consequently, findings related to biomarkers in periodontitis cannot be directly extrapolated to peri-implant diseases (20–23). Therefore, it is necessary to investigate, through prospective studies, the relationship between early postoperative levels of inflammatory cytokines in PICF and the subsequent development of peri-implant diseases. Identifying reliable and minimally invasive predictive biomarkers may provide a scientific basis for early clinical risk stratification, individualized follow-up, and preventive interventions.

## Materials and methods

### Study design and participants

This prospective study included all adult patients who underwent dental implant surgery at the Department of Stomatology, Henan Provincial People’s Hospital between July 2024 and December 2024, for screening. Exclusion criteria were the presence of diabetes mellitus, inability to completely cease smoking, concomitant systemic diseases, poor compliance, and other conditions that could affect study outcomes. Therefore, *a priori* sample size estimation was not performed in the present study.

### Postoperative oral care

Eligible patients received routine postoperative oral care, including oral hygiene instruction, mechanical plaque control, toothbrushing twice daily (morning and evening), and rinsing with water.

### Sample (PICF) collection

At 3 months postoperatively, PICF samples were collected using sterile absorbent paper strips gently inserted into the peri-implant sulcus for 30 s, avoiding contact with blood or saliva to minimize contamination. Collected strips were immediately placed into sterile tubes and stored at −80 °C until analysis. All samples were collected by a single experienced investigator, ensuring consistency and minimizing variability.

### Measurement of inflammatory cytokines

The levels of IL-1β, IL-6, and TNF-*α* in PICF were measured using enzyme-linked immunosorbent assay (ELISA), strictly following the manufacturer’s instructions. Due to the real-world clinical setting and limited sample volumes, cytokine measurements were performed once for each sample rather than in duplicate. Laboratory analyses were conducted according to routine protocols, but not in a fully blinded manner. Cytokine concentrations were expressed in pg./mL.

### Grouping strategy

At 12 months postoperatively, patients were categorized based on the presence or absence of peri-implant mucositis and peri-implantitis, forming the following comparisons: peri-implant mucositis vs. no peri-implant mucositis, and peri-implantitis vs. no peri-implantitis. Intergroup differences in inflammatory cytokine levels measured at 3 months postoperatively were compared. The associations between 3-month PICF cytokine levels and 12-month peri-implant disease outcomes, as well as their potential predictive value, were further analyzed.

### Definition of peri-implant diseases

Peri-implant mucositis (10, 24) was defined according to the 2023 international classification standards and characterized by bleeding on gentle probing (≥1 bleeding point, linear bleeding, or profuse bleeding) and/or suppuration, without bone loss beyond the initial bone remodeling. Peri-implantitis (10, 24) was diagnosed when all of the following criteria were met: (1) bleeding on probing and/or suppuration; (2) an increase in probing depth compared with baseline; and (3) bone loss beyond the initial bone remodeling. In the absence of baseline data, peri-implantitis was defined as bleeding on probing and/or suppuration, probing depth ≥6 mm, and bone loss ≥3 mm.

### Statistical analysis

Continuous variables were expressed as mean ± standard deviation or median (range), depending on their distribution, while categorical variables were presented as counts and percentages. For intergroup comparisons, the chi-square test was used for categorical variables and the Kruskal–Wallis test for continuous variables. Logistic regression analyses were applied to analyze the associations between PICF cytokine levels and the occurrence of peri-implant diseases, with both univariate and multivariate analyses performed. Odds ratios (ORs) and 95% confidence intervals (CIs) were calculated. Receiver operating characteristic (ROC) curves were used to assess the predictive performance of inflammatory cytokines, and the area under the curve (AUC) was determined. All statistical tests were two-sided, with a significance level set at *p* < 0.05. ROC analyses were performed using MedCalc version 19.6.1, AUC plots were generated using GraphPad Prism version 10.1.1, and all other statistical analyses were conducted using SPSS version 29.0.

## Results

### Patient screening and distribution

**Figure 1:**
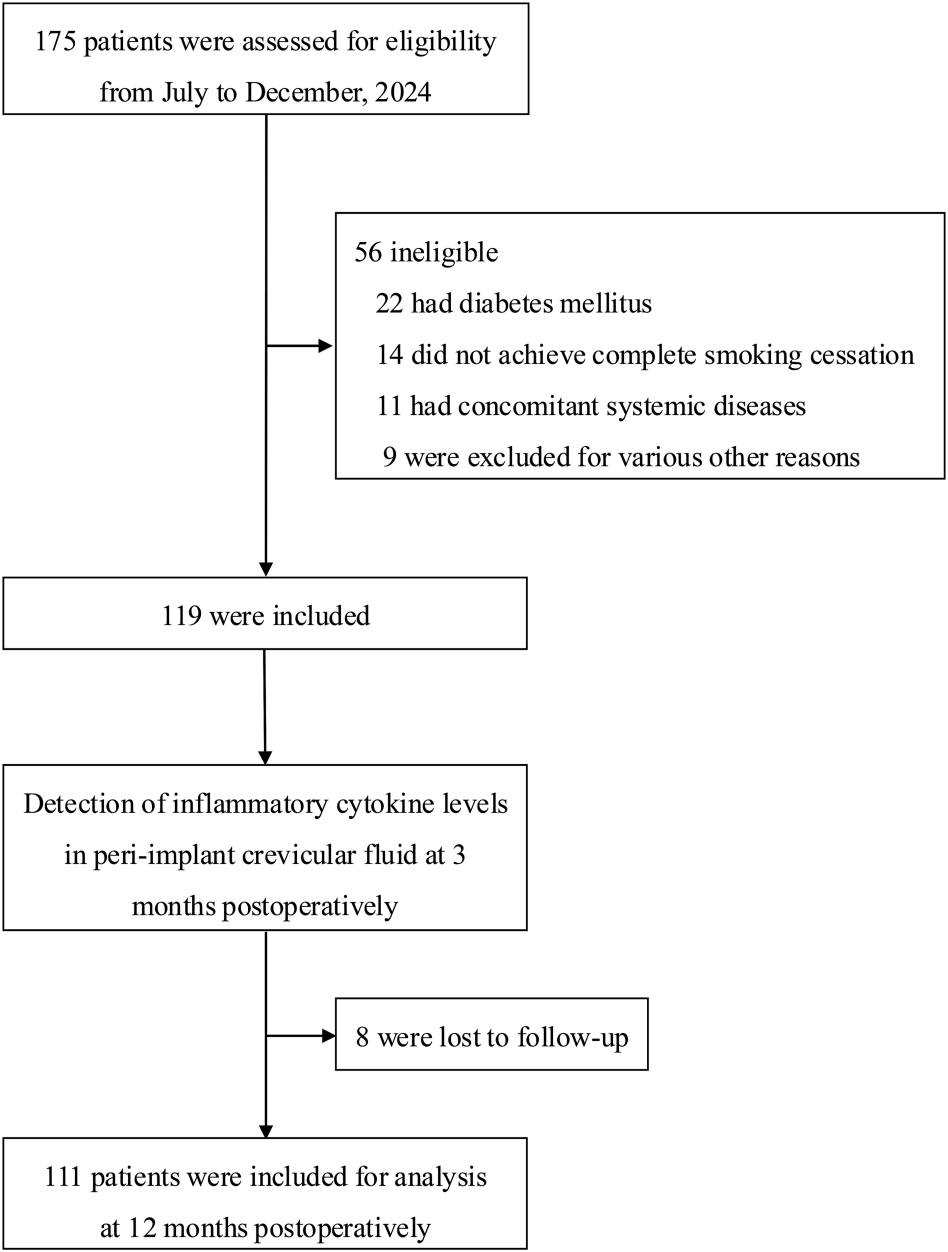
Study flow diagram.

A total of 175 patients met the initial screening criteria. Fifty-six patients were excluded, including 22 with diabetes mellitus, 14 who were unable to completely cease smoking, 11 with other systemic diseases, and 9 for other reasons. Ultimately, 119 patients were enrolled in the study; 8 patients were lost to follow-up before the 12-month postoperative visit. Therefore, 111 patients were included in the final analysis (Figure 1).

### Baseline characteristics of patients

The 111 patients received a total of 157 dental implants. The proportion of male patients was 65.8% (73/111), and the mean age was 60.6 ± 7.1 years (Table 1). The primary causes for tooth loss were similar between groups. Dental caries was the most common cause, followed by periodontal disease, whereas trauma and other causes were relatively uncommon (all *p* > 0.05). Regarding implant placement, the distributions between the maxilla and mandible, as well as between anterior and posterior regions, were comparable between groups (all *p* > 0.05). Most implants were restored using cement-retained prostheses, and single implants were more common than multiple implants. No significant intergroup differences were observed in implant-related characteristics (peri-implant mucositis vs. no peri-implant mucositis, and peri-implantitis vs. no peri-implantitis; all *p* > 0.05). Other relevant baseline characteristics are presented in Table 1.

**Table 1:** Baseline demographical characteristics of patients.

Characteristics	Value (*n* = 111)
Gender (male [%])	73 (65.8)
Age (year)	60.6 ± 7.1
Total implants number	157
Implants number per patient	1.4 ± 0.5
History of periodontitis	31 (27.9)
Main causes for dental implant	
Dental caries	85 (76.6)
Periodontal disease	19 (17.1)
Trauma	2 (1.8)
Others	5 (4.5)
Implant installation	
Maxilla	46 (41.4)
Mandible	65 (58.6)
Anterior	29 (26.1)
Posterior	82 (73.9)
Screw retention	16 (14.4)
Cemented retention	95 (85.6)
Single-unit prosthesis	67 (60.4)
Multiple-unit prosthesis	44 (39.6)

Values in this table are presented as *n*, *n* (%), or mean ± standard deviation. Percentages (%) are calculated based on the total number of patients.

### Inflammatory cytokine levels in PICF at 3 months postoperatively and incidence of peri-implant diseases at 12 months

**Figure 2:**
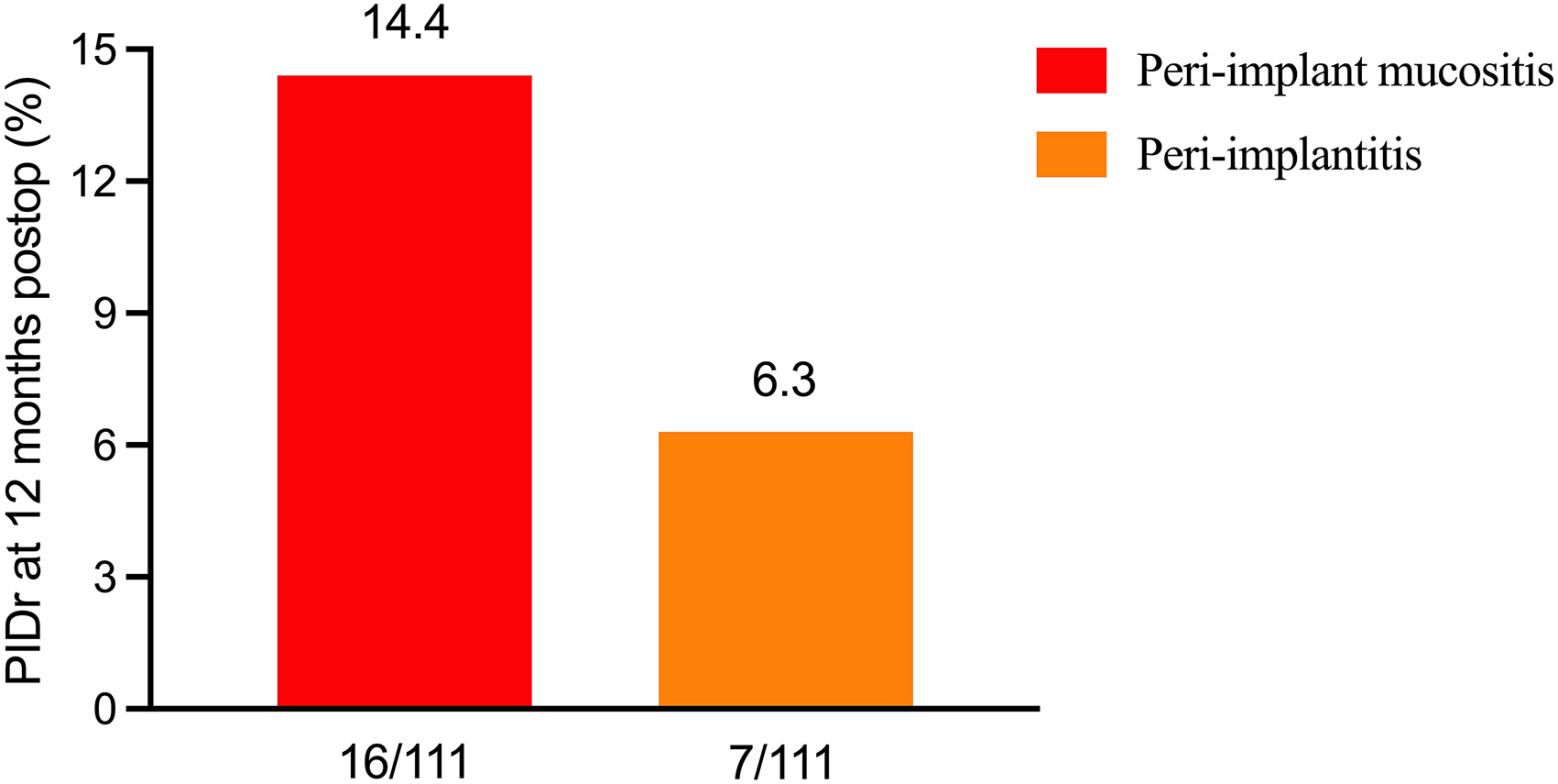
Incidences of peri-implant diseases (PIDr) at 12 months postoperatively (postop).

At 3 months postoperatively, the levels of IL-1β, IL-6, and TNF-*α* in PICF were 20.8 (15, 23.4), 3.7 (2.6, 5.0), and 13.7 (11.8, 16.0) pg./mL, respectively. At 12 months postoperatively, the incidence of peri-implant mucositis was 14.4% (16/111), and the incidence of peri-implantitis was 6.3% (7/111) (Figure 2). It should be specifically noted that five cases lacked baseline data related to the diagnostic criteria for peri-implantitis; however, none of the patients who were ultimately diagnosed with peri-implantitis had missing baseline data.

### Differences in inflammatory cytokine levels

Patients were categorized based on the presence or absence of peri-implant mucositis and peri-implantitis at 12 months postoperatively. For peri-implant mucositis, the levels of IL-1β, IL-6, and TNF-*α* in PICF were 29.0 (21.5, 37.3) vs. 20.3 (13.5, 22.7), 5.0 (3.2, 6.0) vs. 3.6 (2.5, 4.8), and 12.2 (10.8, 16.8) vs. 12.2 (10.8, 16.8) pg./mL, respectively. Significant differences were observed for IL-1β and IL-6 between groups (both *p* < 0.05; Table 2). For peri-implantitis, the corresponding cytokine levels were 29.0 (20.0, 43.0) vs. 20.7 (14.1, 23.1), 5.0 (4.1, 6.0) vs. 3.6 (2.6, 4.9), and 16.1 (13.0, 21.4) vs. 13.6 (11.7, 15.8) pg./mL, respectively. Again, significant intergroup differences were found for IL-1β and IL-6 (both *p* < 0.05; Table 2).

**Table 2:** Differences in peri-implant crevicular fluid inflammatory cytokine levels at 3 months postoperatively among different patient groups at 12 months.

Parameters	Yes	No	*p-*value
Peri-implant mucositis			
Interleukin-1β (pg/mL)	29.0 (21.5, 37.3)	20.3 (13.5, 22.7)	<0.001
Interleukin-6 (pg/mL)	5.0 (3.2, 6.0)	3.6 (2.5, 4.8)	0.005
Tumor necrosis factor-α (pg/mL)	12.2 (10.8, 16.8)	13.8 (12.0, 15.9)	0.356
Peri-implantitis			
Interleukin-1β (pg/mL)	29.0 (20.0, 43.0)	20.7 (14.1, 23.1)	0.007
Interleukin-6 (pg/mL)	5.0 (4.1, 6.0)	3.6 (2.6, 4.9)	0.013
Tumor necrosis factor-α (pg/mL)	16.1 (13.0, 21.4)	13.6 (11.7, 15.8)	0.079

Values in this table are presented as median and interquartile ranges.

### Univariate logistic regression analysis

Each inflammatory cytokine (IL-1β, IL-6, and TNF-α) was entered separately into univariate logistic regression analysis. IL-1β and IL-6 were significantly associated with peri-implant mucositis (both *p* < 0.05), whereas TNF-α was not (Table 3). Interestingly, IL-1β, IL-6, and TNF-α were all significantly associated with peri-implantitis (all *p* < 0.05; Table 3).

**Table 3:** Unadjusted univariate logistic regression analysis of peri-implant diseases at 12 months postoperatively.

Parameters	UOR (95% CI)	*β* coefficient	*P-*value
Peri-implant mucositis			
Interleukin-1β (pg/mL)	1.3 (1.1–1.6)	0.292	<0.001
Interleukin-6 (pg/mL)	1.9 (1.2–2.9)	0.624	0.005
Tumor necrosis factor-α (pg/mL)	–	–	0.475
Peri-implantitis			
Interleukin-1β (pg/mL)	1.2 (1.1–1.4)	0.194	<0.001
Interleukin-6 (pg/mL)	2.4 (1.2–4.8)	0.858	0.017
Tumor necrosis factor-α (pg/mL)	1.5 (1.1–2.0)	0.381	0.010

CI, confidence interval; UOR, unadjusted odds ratio.

### Multivariate logistic regression analysis

When IL-1β and IL-6 were simultaneously included in the multivariate analysis for peri-implant mucositis, only IL-1β remained significantly associated with peri-implant mucositis (*p* < 0.05), whereas IL-6 did not (Table 4). Similarly, when IL-1β, IL-6, and TNF-*α* were simultaneously included in the multivariate analysis for peri-implantitis, only IL-1β was associated with peri-implantitis (*p* < 0.05), while IL-6 and TNF-α were not (Table 4).

**Table 4:** Adjusted multivariate logistic regression analysis of peri-implant diseases at 12 months postoperatively.

Parameters	UOR (95% CI)	*β* coefficient	*P-*value
Peri-implant mucositis			
Interleukin-1β (pg/mL)	1.3 (1.1–1.5)	0.273	<0.001
Interleukin-6 (pg/mL)	-	-	0.056
Peri-implantitis			
Interleukin-1β (pg/mL)	1.2 (1.0–1.3)	0.166	0.011
Interleukin-6 (pg/mL)	–	–	0.101
Tumor necrosis factor-α (pg/mL)	–	–	0.141

CI, confidence interval; AOR, adjusted odds ratio.

### ROC curve analysis

**Figure 3:**
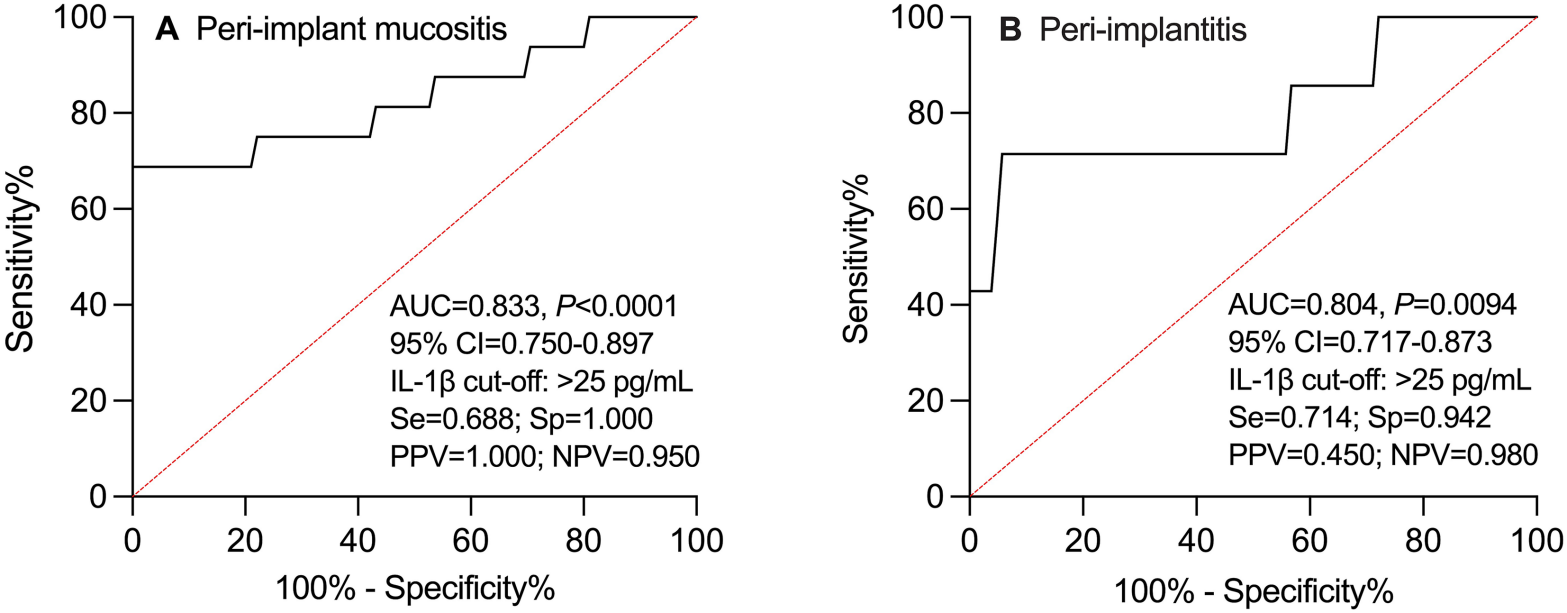
ROC curve of IL-1β in PICF at 3 months postoperatively as a potential predictor of peri-implant mucositis **(A)** and peri-implantitis **(B)** at 12 months. ROC, receiver operating characteristic; IL-1β, interleukin-1β; PICF, peri-implant crevicular fluid; AUC, area under the curve; 95% CI, 95% confidence interval; Se, sensitivity; Sp, specificity; PPV, positive predictive value; NPV, negative predictive value.

The ROC curve analysis suggested a potential cutoff value of >25 pg./mL for IL-1β in predicting peri-implant diseases. A PICF IL-1β level >25 pg./mL at 3 months postoperatively was associated with an AUC of 0.833 (*p* < 0.0001) for predicting peri-implant mucositis at 12 months and an AUC of 0.804 (*p* = 0.0094) for predicting peri-implantitis at 12 months. These findings indicate a possible discriminatory ability; however, this cutoff was derived from the current dataset and should be considered exploratory and hypothesis-generating rather than validated (Figure 3).

## Discussion

From the perspective of disease progression, peri-implant mucositis may progress to peri-implantitis if not effectively controlled in a timely manner, leading to marginal bone loss and even implant failure (1, 2, 10, 11). Therefore, identifying simple, low-cost, and highly compliant measures to reduce the incidence of peri-implant mucositis during the early stage after implant rehabilitation is of considerable clinical importance. The present study demonstrated that elevated levels of IL-1β in PICF at 3 months postoperatively may serve as a potential predictor of peri-implant disease at 12 months, suggesting a potential clinical value of IL-1β in early risk assessment and even intervention for peri-implant diseases in the future.

In this study, those who developed peri-implant diseases exhibited significantly higher levels of IL-1β and IL-6 in PICF than those without diseases. However, further multivariate analyses revealed that only IL-1β remained significantly associated with diseases occurrence, whereas the statistical significance of IL-6 disappeared. This finding suggests that IL-6 may primarily reflect the magnitude of the inflammatory response, and its role in disease development may depend on IL-1β–mediated inflammatory pathways (7, 25–28), thereby failing to demonstrate potential predictive value. The discrepancy between univariate and multivariate analyses underscores the importance of accounting for potential collinearity among inflammatory mediators. Pro-inflammatory cytokines often function within interconnected signaling networks rather than acting independently. When evaluated individually, multiple cytokines may show statistical associations with disease; however, when analyzed simultaneously, the dominant upstream mediator may retain significance while downstream or correlated markers lose potential predictive value. In this context, IL-1β may represent the principal inflammatory driver captured in the present study.

Notably, TNF-α was associated with peri-implantitis in univariate analysis but lost statistical significance after multivariate adjustment. This result is consistent with previous reports showing no significant association (6, 29) but contrasts with another study reporting a significant association (30). Moreover, different inflammatory mediators may act at different stages of the inflammatory response, and TNF-*α* may be more reflective of inflammatory activity rather than serving as a key predictor of disease onset. Furthermore, the relatively low incidence of peri-implantitis in the present cohort may have limited the statistical stability of multivariate estimates. Future studies with larger sample sizes and higher event numbers are warranted to further clarify the potential contribution of TNF-α and other cytokines to disease progression.

The ROC curve analysis further suggested the possibility of IL-1β as a potential predictor of peri-implant diseases. Using a cutoff value of >25 pg./mL for PICF IL-1β at 3 months postoperatively, a moderate-to-good discriminative ability was observed within this dataset for predicting peri-implant mucositis and peri-implantitis at 12 months. These findings suggest that early postoperative assessment of inflammatory status may facilitate risk stratification, thereby aiding in the identification of high-risk patients and optimization of maintenance frequency and timing of preventive interventions during follow-up.

However, several limitations of this study should be acknowledged. First, although this was a prospective single-center study, the sample size—particularly the number of peri-implantitis cases—was relatively small, and no *a priori* power calculation was performed. The final cohort was determined by consecutively eligible patients within the study period, which may have limited statistical power, especially in multivariate analyses, and affected the generalizability of the findings. Second, certain methodological factors may have introduced measurement variability. Inflammatory cytokines in PICF were assessed at a single postoperative time point, and although standardized sampling procedures were applied, inherent uncertainties related to crevicular fluid collection (e.g., local fluid volume variation or micro-contamination) cannot be completely excluded. In addition, some patients received multiple implants, and analyses were conducted at the patient level without hierarchical modeling to account for potential intra-patient clustering effects. This may affect the precision of statistical estimates, and should be considered a methodological limitation of the study. Third, several potentially relevant clinical parameters were not systematically recorded, including peri-implant soft tissue phenotype (e.g., keratinized mucosa width and tissue thickness) and plaque control–related indices. These local factors may influence inflammatory responses and cytokine expression, and their absence limits more comprehensive adjustment for confounding. Fourth, the use of the ELISA method may introduce technical variability and potential measurement errors, which should be considered when interpreting the results. In addition, formal reliability testing of the measurement procedures was not performed, which may affect the reproducibility of the findings. Finally, the follow-up period was limited to 12 months postoperatively, reflecting early peri-implant disease occurrence only. Despite these limitations, our preliminary findings provide valuable insights into early inflammatory changes around implants and suggest that IL-1β in PICF may serve as a potential biomarker for peri-implant diseases risk.

## Conclusion

In summary, the present findings suggest that elevated IL-1β levels in PICF at 3 months postoperatively may be associated with the occurrence of peri-implant diseases at 12 months. These results indicate a potential role for early inflammatory risk assessment. However, longer-term studies with repeated biomarker measurements and more comprehensive clinical characterization are warranted to validate and extend the present findings.

## Data Availability

The raw data supporting the conclusions of this article will be made available by the authors, without undue reservation.
